# The Impact of CC16 on Pulmonary Epithelial-Driven Host Responses during *Mycoplasma pneumoniae* Infection in Mouse Tracheal Epithelial Cells

**DOI:** 10.3390/cells12151984

**Published:** 2023-08-01

**Authors:** Natalie Iannuzo, Alane Blythe C. Dy, Stefano Guerra, Paul R. Langlais, Julie G. Ledford

**Affiliations:** 1 Department of Cellular and Molecular Medicine, University of Arizona, Tucson, AZ 85724, USA; niannuzo@arizona.edu; 2 Asthma and Airway Disease Research Center, Tucson, AZ 85724, USA; 3 Department of Medicine, Division of Endocrinology, University of Arizona, Tucson, AZ 85724, USA

**Keywords:** CC16, *Mycoplasma pneumoniae*, airway epithelium, mass spectrometry, quantitative proteomics

## Abstract

Club Cell Secretory Protein (CC16) plays many protective roles within the lung; however, the complete biological functions, especially regarding the pulmonary epithelium during infection, remain undefined. We have previously shown that CC16-deficient (CC16^−/−^) mouse tracheal epithelial cells (MTECs) have enhanced Mp burden compared to CC16-sufficient (WT) MTECs; therefore, in this study, we wanted to further define how the pulmonary epithelium responds to infection in the context of CC16 deficiency. Using mass spectrometry and quantitative proteomics to analyze proteins secreted apically from MTECs grown at an air–liquid interface, we investigated the protective effects that CC16 elicits within the pulmonary epithelium during *Mycoplasma pneumoniae* (Mp) infection. When challenged with Mp, WT MTECs have an overall reduction in apical protein secretion, whereas CC16^−/−^ MTECs have increased apical protein secretion compared to their unchallenged controls. Following Gene Ontology and Kyoto Encyclopedia of Genes and Genomes (KEGG) assessment, many of the proteins upregulated from CC16^−/−^ MTECS (unchallenged and during Mp infection) were related to airway remodeling, which were not observed by WT MTECs. These findings suggest that CC16 may be important in providing protection within the pulmonary epithelium during respiratory infection with Mp, which is the major causative agent of community-acquired pneumoniae.

## 1. Introduction

CC16, a member of the secretoglobin family of disulfide-linked dimeric proteins, is encoded by the *SCGB1A1* gene and secreted by club cells and non-ciliated epithelial cells in the pulmonary epithelium [1,2,3]. Although CC16 is one of the most abundant proteins in bronchoalveolar lavage fluid, the complete biological functions of this protein, especially in regard to the pulmonary epithelium, remain incompletely defined [4]. Despite the lack of mechanistic understanding, studies have shown that CC16 plays a role in mediating anti-inflammatory [5,6,7,8] and antioxidant [1,4,7,9] responses in the lung. Additionally, CC16′s potent phospholipase A2 inhibitory activity [3,10,11], ability to suppress pro-inflammatory cytokine expression [12,13,14], and capacity to bind polychlorinated biphenyls [1,15] and retinols [2,3] further confirms its role in controlling pulmonary airway inflammation and oxidative stress.

Mp is an “atypical” bacteria that can cause both upper and lower respiratory tract infections as well as pneumonia [16]. Mp is the major causative agent of community-acquired pneumonia, and it is estimated that there are 2 million Mp cases annually, 100,000 of which result in hospitalizations in the United States [16,17]. The severity of Mp infections is related to the degree by which the host immune system responds to infection [18]. During infection, Mp attaches to the host’s respiratory epithelium and produces many cytotoxic proteins, thereby protecting itself from removal by mucociliary escalator mechanisms while damaging the host’s pseudostratified epithelium [18,19].

Our group has shown that in WT mice, CC16 plays a protective role during early life Mp infections in mice by limiting inflammation and lung remodeling. Moreover, mice lacking CC16 during early life Mp infection have severely augmented airway hyperresponsiveness and collagen deposition, which results in decreased lung function in adulthood [20]. We have also shown that CC16 protects against pulmonary leukocyte infiltration and inflammation during Mp infection by binding to the integrin complex Very Late Antigen-4 (VLA-4) on leukocytes [21]. Additionally, we found that CC16^−/−^ MTECs have increased Mp burden when infected in vitro compared to WT MTECs (CC16 sufficient), indicating an epithelial-driven impairment in host defense during infections [20]. Based on these supporting data, and since Mp targets the pulmonary epithelium during infection, we seek to define how CC16^−/−^ MTECs respond when challenged with Mp. Clinically, for patients with known low CC16 levels, such as asthma, chronic obstructive pulmonary disease (COPD), and cystic fibrosis patients [5,22,23,24,25,26,27,28,29,30], an impairment in host responses would likely result in increased respiratory infections, inflammation, and remodeling.

## 2. Results

### 2.1. WT MTECs Have Decreased Apical Proteins Secretions during Mp Infection

**Figure 1:**
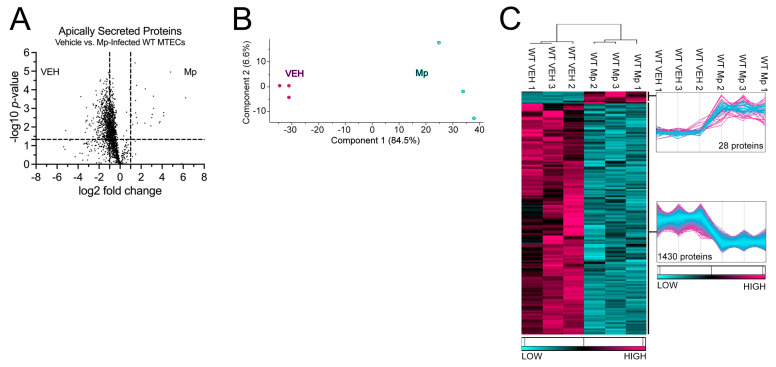
Mp infection decreases secretion of apical proteins in WT MTECs. (**A**) A volcano plot of the apically secreted proteins from WT MTECs treated with media (vehicle) or infected with Mp for 48 h. The horizontal black line represents the cut–off for a *p* value of <0.05, while the two vertical lines represent the cut–off values of 2–fold change in either the positive or negative direction. Proteins with decreased expression by Mp infection have a negative fold change, while proteins with increased expression by Mp infection have a positive fold change. (**B**) Unbiased principal component analysis (PCA) of the 1458 secreted proteins during vehicle treatment and Mp infection shows consistency among the individual biological samples within each group. (**C**) Unbiased hierarchical clustering of the 1458 proteins secreted during vehicle treatment and Mp infection confirmed that the different treatment groups clustered together. *n* = 3 per group.

WT MTECs were infected with Mp for 48 h, after which apically secreted proteins from vehicle-treated and Mp-infected WT MTECs were identified and characterized using mass spectrometry and quantitative proteomics, respectively. To obtain a global picture of protein expression upon vehicle treatment and Mp infection, apically secreted proteins were graphed in a volcano plot to identify significant proteins (2-way ANOVA analysis; *p* < 0.05) with a fold change > 2 (Figure 1A). This analysis identified 1706 total apically secreted proteins from vehicle-treated and Mp-infected WT MTECs across six biological replicates. All significant proteins were graphed in a heat map to examine protein expression changes between treatment groups for the WT MTECs (Figure 1C). Of the 1458 differentially secreted proteins that were statistically different between treatment groups, Mp infection resulted in an increase in 28 proteins and a decrease in 1430 proteins by the WT MTECs (Figure 1C). Unbiased principal component analysis (PCA) of the 1458 differentially secreted proteins demonstrated consistency among the samples within each treatment group (Figure 1B) [31].

### 2.2. WT MTECs Have Decreased Secretion of Antioxidant Proteins during Mp Infection

**Figure 2:**
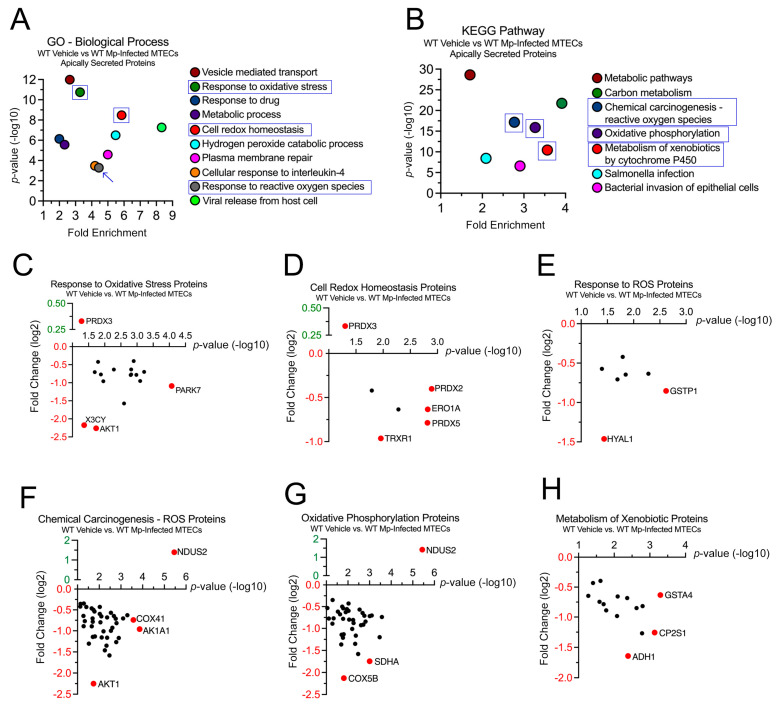
WT MTECs have decreased secretion of antioxidant proteins during Mp infection. Scatter plots of the Gene Ontology (GO)—Biological Process and KEGG Pathway enrichment findings for the proteins with significantly increased and decrease expression during Mp infection. GO and KEGG terms of interest are highlighted in blue (**A**,**B**). All proteins corresponding to the highlighted GO and KEGG enrichment terms—“Response to Oxidative Stress” (**C**), “Cell Redox Homeostasis” (**D**), “Response to ROS” (**E**), “Chemical Carcinogenesis—ROS” (**F**), “Oxidative Phosphorylation” (**G**), and “Metabolism of Xenobiotics” (**H**)—were graphed to determine expression. Proteins of interest are highlighted in red and labeled.

Next, we sought to examine the 1458 proteins differentially secreted by the WT MTECs during vehicle (control) and Mp infection to better understand the impact of CC16 on pulmonary epithelial-driven responses. In the GO–Biological Process analysis, 19 proteins were associated with “Response to Oxidative Stress” (*p* = 3.65 × 10^−4^), 17 proteins were associated with “Cell Redox Homeostasis” (*p* = 3.32 × 10^−9^), and 9 proteins were associated with “Response to Reactive Oxygen Species” (*p* = 5.20 × 10^−4^) (Figure 2A). Fold changes for individual proteins were normalized to vehicle treatment; therefore, proteins with increased expression during Mp infection had a positive fold change, while proteins with decreased expression during Mp infection had a negative fold change. The proteins with the greatest significance or fold change for “Response to Oxidative Stress” are Parkinson Disease Protein 7 (PARK7) and RAC-alpha serine/threonine protein kinase (AKT1), respectively (Figure 2C). The proteins with the greatest significance or fold change for “Cell Redox Homeostasis” are Peroxiredoxin-2 (PRDX2) and Thioredoxin Reductase 1(TRXR1), respectively (Figure 2D). The proteins with the greatest significance or fold change for “Response to Reactive Oxygen Species” are Glutathione S-transferase P 1 (GSTP1) and Hyaluronidase-1 (HYAL1), respectively (Figure 2E). Peroxiredoxin-3 (PRDX3) had increased expression during Mp infection and was associated with both “Response to Oxidative Stress” and Cell Redox Homeostasis” (Figure 2C,D).

In the KEGG pathway analysis, 78 proteins were associated with “Chemical Carcinogenesis—Reactive Oxygen Species” (*p* = 6.86 × 10^−18^), 56 proteins were associated with “Oxidative Phosphorylation” (*p* = 1.27 × 10^−16^), and 33 proteins were associated with “Metabolism of Xenobiotics by Cytochrome P450” (*p* = 3.60 × 10^−11^) (Figure 2B). The proteins with the greatest significance or fold change for “Chemical Carcinogenesis—Reactive Oxygen Species” are NADH Dehydrogenase Iron-Sulfur Protein 2 (NDUS2) and AKT1, respectively (Figure 2F). The proteins with the greatest significance or fold change for “Oxidative Phosphorylation” are NDUS2 and Cytochrome C Oxidase Subunit 5B (COX5B), respectively (Figure 2G). The proteins with the greatest significance or fold change for “Metabolism of Xenobiotics by Cytochrome P450” are Glutathione S-Transferase A4 (GSTA4) and Alcohol Dehydrogenase 1 (ADH1), respectively (Figure 2H). NDUS2 had increased expression during Mp infection and was associated with both “Chemical Carcinogenesis—Reactive Oxygen Species” and “Oxidative Phosphorylation” (Figure 2F,G).

### 2.3. CC16^−/−^ MTECs Have Increased Apical Protein Secretions during Mp Infection

**Figure 3:**
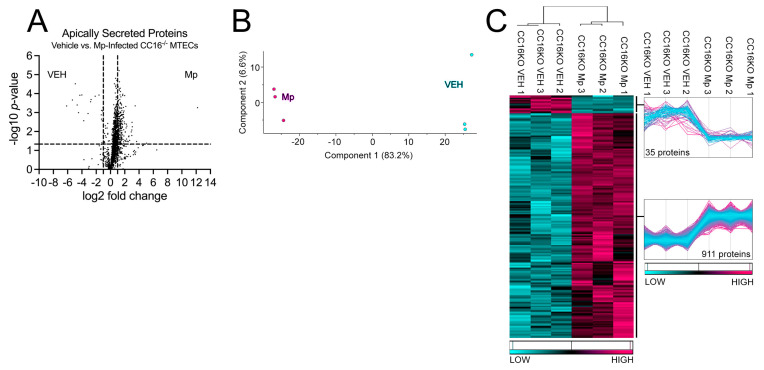
CC16^−/−-^ MTECs have increased apical protein secretions during Mp infection. (**A**) A volcano plot of the apically secreted proteins from CC16^−/−^ MTECs treated with media (vehicle) or infected with Mp for 48 h. The horizontal black line represents the cut–off for a *p* value of <0.05, while the two vertical lines represent the cut–off values of 2–fold change in either the positive or negative direction. Proteins with decreased expression by Mp infection have a negative fold change, while proteins with increased expression by Mp infection have a positive fold change. (**B**) Unbiased principal component analysis (PCA) of the 946 significantly secreted proteins during vehicle treatment and Mp infection shows consistency among the individual biological samples within each group. (**C**) Unbiased hierarchical clustering of the 946 proteins secreted during vehicle treatment and Mp infection confirmed that the different treatment groups clustered together. *n* = 3 per group.

Next, we sought to determine how CC16 deficiency impacts pulmonary epithelial-driven responses during Mp infection. Apically secreted proteins from vehicle-treated and Mp-infected CC16^−/−^ MTECs were identified and characterized using mass spectrometry and quantitative proteomics, respectively. To obtain a global picture of protein expression upon vehicle treatment and Mp infection, apically secreted proteins were graphed in a volcano plot to identify significant proteins (2-way ANOVA analysis; *p* < 0.05) with a fold change > 2 (Figure 3A). This analysis identified 1707 total apically secreted proteins from vehicle-treated and Mp-infected CC16^−/−^ MTECs across six biological replicates. All significant proteins were graphed in a heat map to look at protein expression changes between treatment groups for the CC16^−/−^ MTECs (Figure 3C). Of the 946 significantly secreted proteins, Mp infection resulted in an increase in 911 proteins and a decrease in 35 proteins by the CC16^−/−^ MTECs (Figure 3C). Unbiased principal component analysis (PCA) of the 946 differentially secreted proteins demonstrated consistency among the samples within each treatment group (Figure 3B) [31].

### 2.4. CC16^−/−^ MTECs Have Increased Secretion of Airway Remodeling and Bacterial Invasion Proteins during Mp Infection

**Figure 4:**
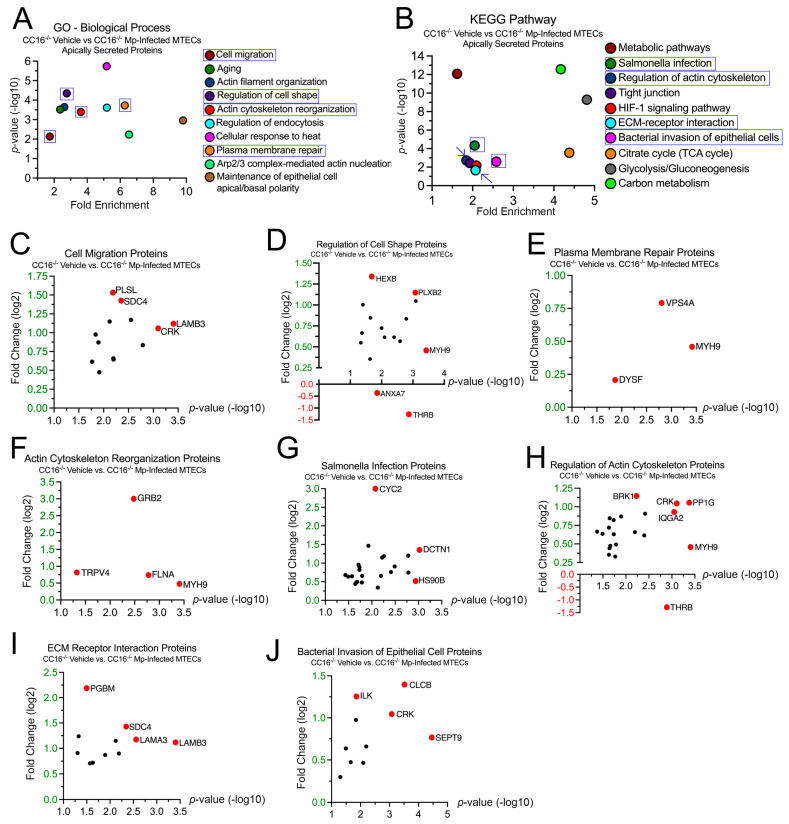
CC16^−/−^ MTECs have increased secretion of airway remodeling and bacterial invasion proteins during Mp infection. Scatter plots of the Gene Ontology (GO)—Biological Process and KEGG pathway enrichment findings for the proteins with significantly increased secretion by the CC16^−/−^ MTECs during vehicle treatment and Mp infection. GO and KEGG terms of interest are highlighted in blue (**A**,**B**). All proteins corresponding to the highlighted GO and KEGG enrichment terms—“Cell Migration” (**C**), “Regulation of Cell Shape” (**D**), “Plasma Membrane Repair” (**E**), “Actin Cytoskeleton Reorganization” (**F**), “Salmonella Infection” (**G**), “Regulation of Actin Cytoskeleton” (**H**), “ECM Receptor Interaction” (**I**) and “Bacterial Invasion of Epithelial Cell” (**J**)—were graphed to determine expression. Proteins of interest are highlighted in red and labeled.

To better understand how CC16 deficiency impacts pulmonary epithelial cells during Mp infection, we examined the 946 proteins whose secretion was significantly increased from the CC16^−/−^ MTECs. In the GO–Biological Process analysis, 27 proteins were associated with “Cell Migration” (*p* = 7.37 × 10^−3^), 22 proteins were associated with “Regulation of Cell Shape” (*p* = 4.48 × 10^−5^), 8 proteins were associated with “Plasma Membrane Repair” (*p* = 1.87 × 10^−4^), and 12 proteins were associated with “Actin Cytoskeleton Reorganization” (*p* = 4.06 × 10^−4^) (Figure 4A). Fold changes for individual proteins were normalized to vehicle treatment; therefore, proteins with increased expression during Mp infection had a positive fold change, while proteins with increased expression during vehicle treatment had a negative fold change. The proteins with the greatest significance or fold change for “Cell Migration” are Laminin Subunit Beta-3 (LAMB3) and Plastin-2 (PLSL), respectively (Figure 4C). The proteins with the greatest significance or fold change for “Regulation of Cell Shape” are Myosin-9 (MYH9) and Prothrombin (THRB), respectively (Figure 3D). THRB had increased expression during vehicle treatment; therefore, THRB levels were decreased during Mp infection in the absence of CC16. The proteins with the greatest significance or fold change for “Plasma Membrane Repair” are MYH9 and Vacuolar Protein Sorting-Associated Protein 4A (VPS4A), respectively (Figure 4E). The proteins with the greatest significance or fold change for “Actin Cytoskeleton Reorganization” are MYH9 and Growth Factor Receptor-Bound Protein 2 (GRB2), respectively (Figure 4F).

In the KEGG analysis, 37 proteins were associated with “Salmonella Infection” (*p* = 4.67 × 10^−5^), 29 proteins were associated with “Regulation of Actin Cytoskeleton” (*p* = 1.88 × 10^−3^), 13 proteins were associated with “ECM–Receptor Interaction” (*p* = 2.16 × 10^−2^), and 14 proteins were associated with “Bacterial Invasion of Epithelial Cells” (*p* = 2.53 × 10^−3^) (Figure 4B). The proteins with the greatest significance or fold change for “Salmonella Infection” are Dynactin Subunit 1 (DCTN1) and Cytochrome C (CYC2), respectively (Figure 2G). The proteins with the greatest significance or fold change for “Regulation of Actin Cytoskeleton” are MYH9 and THRB, respectively (Figure 2H). The proteins with the greatest significance or fold change for “ECM–Receptor Interaction” are Laminin Subunit Beta-3 (LAMB3) and Basement Membrane-Specific Heparin Sulfate Proteoglycan Core Protein (PGBM), respectively (Figure 2I). The proteins with the greatest significance or fold change for “Bacterial Invasion of Epithelial Cells” are Septin-9 (SEPT9) and Clathrin Light Chain B (CLCB), respectively (Figure 2J).

### 2.5. CC16 Mediates Pulmonary Epithelial Cell Apical Protein Secretion during Mp Infection

**Figure 5:**
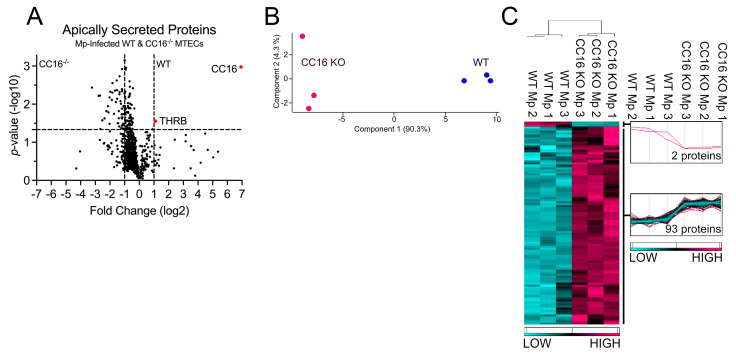
WT MTECs have decreased secretion of apical proteins during Mp infection. (**A**) A volcano plot of the apically secreted proteins from WT and CC16^−/−^ MTECs infected with Mp for 48 h. The horizontal black line represents the cut–off for a *p* value of <0.05, while the two vertical lines represent the cut–off values of 2–fold change in either the positive or negative direction. Proteins with increased expression by CC16^−/−^ MTECs treatment have a negative fold change, while proteins with increased expression by WT MTECs have a positive fold change. (**B**) Unbiased principal component analysis (PCA) of the 95 differentially secreted proteins during Mp infection shows consistency among the individual biological samples within each group. (**C**) Unbiased hierarchical clustering of the 95 proteins secreted during Mp treatment confirmed that the different treatment groups clustered together. *n* = 3 per group.

Next, we compared proteins secreted by both WT and CC16^−/−^ MTECs during Mp infection to better understand how CC16 impacts pulmonary epithelial-driven responses during Mp infection. Based on our previous experiments showing that CC16^−/−^ mice infected with Mp have increased airway remodeling, compared to infected WT mice [20,21], we sought to determine if during Mp infection, CC16-sufficient (WT) MTECs would be protected from epithelial-driven airway remodeling responses compared to CC16^−/−^ MTECs. Additionally, based on prior publications, we expected that WT MTECs would have an increased expression of antioxidants during Mp infection, which may assist in attenuating the Mp pathogen burden, as previously observed [20]. Apically secreted proteins from Mp-infected WT and CC16^−/−^ MTECs were identified and characterized using mass spectrometry and quantitative proteomics, respectively. To obtain a global picture of protein expression upon Mp infection, apically secreted proteins were graphed in a volcano plot to identify significant proteins (2-way ANOVA analysis; *p* < 0.05) with a fold change > 2 (Figure 5A). This analysis identified 937 total apically secreted proteins from Mp-infected WT and CC16^−/−^ MTECs across six biological replicates. The proteins that were deemed significant from the volcano plot were graphed in a heat map to look at protein expression changes between WT and CC16^−/−^ MTECs (Figure 5C). Of the 95 differentially secreted proteins during Mp infection, only two proteins were secreted more by WT MTECs, while 93 proteins were secreted more by CC16^−/−^ MTECs (Figure 1C). The two identified proteins by the WT MTECs were CC16 and THRB (Thrombin) and are labeled and highlighted red in the volcano plot (Figure 5A). Unbiased principal component analysis (PCA) of the 95 significantly secreted proteins demonstrated consistency among the samples within each treatment group (Figure 5B) [31].

### 2.6. CC16 Deficiency Increases Pulmonary Epithelial-Driven Airway Remodeling during Mp Infection

**Figure 6:**
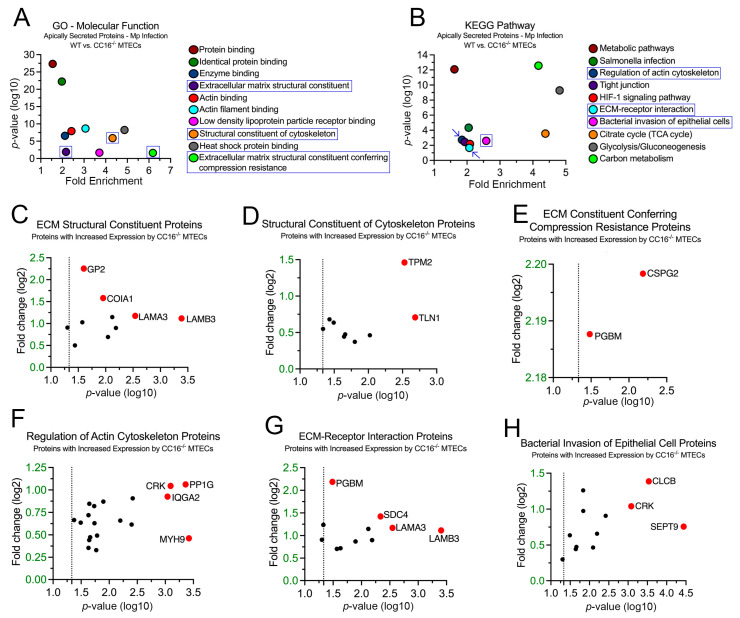
CC16^−/−^ MTECs have increased secretion of proteins related to airway remodeling during Mp infection. Scatter plots of the Gene Ontology (GO)–Molecular Function (**A**) and KEGG pathway (**B**) enrichment findings for the proteins with significantly increased expression by the CC16^−/−^ MTECs during Mp infection. GO and KEGG enrichment terms of interest are highlighted in blue. All proteins corresponding to the highlighted GO and KEGG enrichment terms—“ECM Structural Constituent” (**C**), “Structural Constituent of Cytoskeleton” (**D**), “ECM Conferring Compression Resistance” (**E**), “Regulation of Actin Cytoskeleton (**F**), “ECM–Receptor Interaction” (**G**), and “Bacterial Invasion of Epithelial Cell” (**H**)—were graphed to determine expression. Proteins of interest are highlighted in red and labeled.

Next, we sought to examine the 93 proteins whose expression was significantly increased in the CC16^−/−^ MTECs to better understand how Mp infection impacts pulmonary epithelial-driven responses. In the GO–Molecular Function analysis, 10 proteins were associated with “Extracellular Matrix Structural Constituent” (*p* = 1.25 × 10^−2^), 9 proteins were associated with “Structural Constituent of Cytoskeleton Proteins” (*p* = 1.29 × 10^−6^), and 2 proteins were associated with “Extracellular Matrix Structural Constituent Conferring Compression Resistance” (*p* = 2.37 × 10^−2^) (Figure 6A,C–E). The proteins with the greatest fold change or significance for “Extracellular Matrix Structural Constituent” are Glycoprotein 2 (GP2), Collagen Type XVIII Alpha 1 Chain (COIA1), Laminin Subunit Alpha 3 (LAMA3), and Laminin Subunit Beta 3 (LAMB3) (Figure 6C). The proteins with the greatest fold change or significance for “Structural Constituent of Cytoskeleton Proteins” are Tropomyosin 2 (TPM2) and Talin 1 (TLN1) (Figure 6D). The proteins with the greatest fold change or significance for “Extracellular Matrix Structural Constituent Conferring Compression Resistance” are Versican (CSPG2) and PGBM (Figure 6E). The abundance of enrichment terms associated with extracellular matrix components for the CC16^−/−^ MTECs during Mp infection may represent increased airway remodeling, which aligns with previous findings [20].

In the KEGG pathway analysis, 19 proteins were associated with “Regulation of Actin Cytoskeleton” (*p* = 1.89 × 10^−3^), 12 proteins were associated with “ECM–Receptor Interaction” (*p* = 2.16 × 10^−2^), and 12 proteins were associated with “Bacterial Invasion of Epithelial Cells” (*p* = 2.53 × 10^−3^) (Figure 6B,F–H). The proteins with the greatest fold change or significance for “Regulation of Actin Cytoskeleton” are Adapter Molecule CRK (CRK), Serine/Threonine-Protein Phosphatase PP1-Gamma Catalytic Subunit (PP1G), Ras GTPase-Activating-Like Protein (IQGA2), and MYH9 (Figure 6F). The proteins with the greatest fold change or significance for “ECM–Receptor Interaction Proteins” are PGBM, Syndecan-4 (SDC4), LAMA3, and LAMB3 (Figure 5G). The proteins with the greatest fold change or significance for “Bacterial Invasion of Epithelial Cells” are CRK, CLCB, and SEPT9 (Figure 6H). LAMA3, LAMB3, and PGMB, which are associated with the extracellular matrix, were discovered in both the GO–Molecular Function and KEGG analyses.

Since most of the proteins upregulated by the CC16^−/−^ MTECs during Mp infection may be associated with airway remodeling and bacterial invasion, we used a human eQTL database (QTLbase) [32] to associate SNPs in genes within lung tissue encoding for these protein to their expression changes (Table 1). From this analysis, we were able to identify risk alleles within genes encoding for the ECM, cell migration, and cytoskeletal proteins, which may be important for airway remodeling, lung function, and Mp pathogenesis. To explore whether these variants were associated with respiratory-related phenotypes, we selected SNPs from Table 1 that were associated with a predicted increase in gene expression and searched publicly available datasets to look at their associations with asthma (GCST010043) [33] and FEV_1_ (GCST00743) [34]. We identified one gene, *GRB2*, that had variants that were significantly associated (*p* < 0.05) with at least one of these two phenotypes (Table 2).

### 2.7. Validation of Apically Secreted Proteins in MTECs and Mouse Lung Tissue by Western Blot

**Figure 7:**
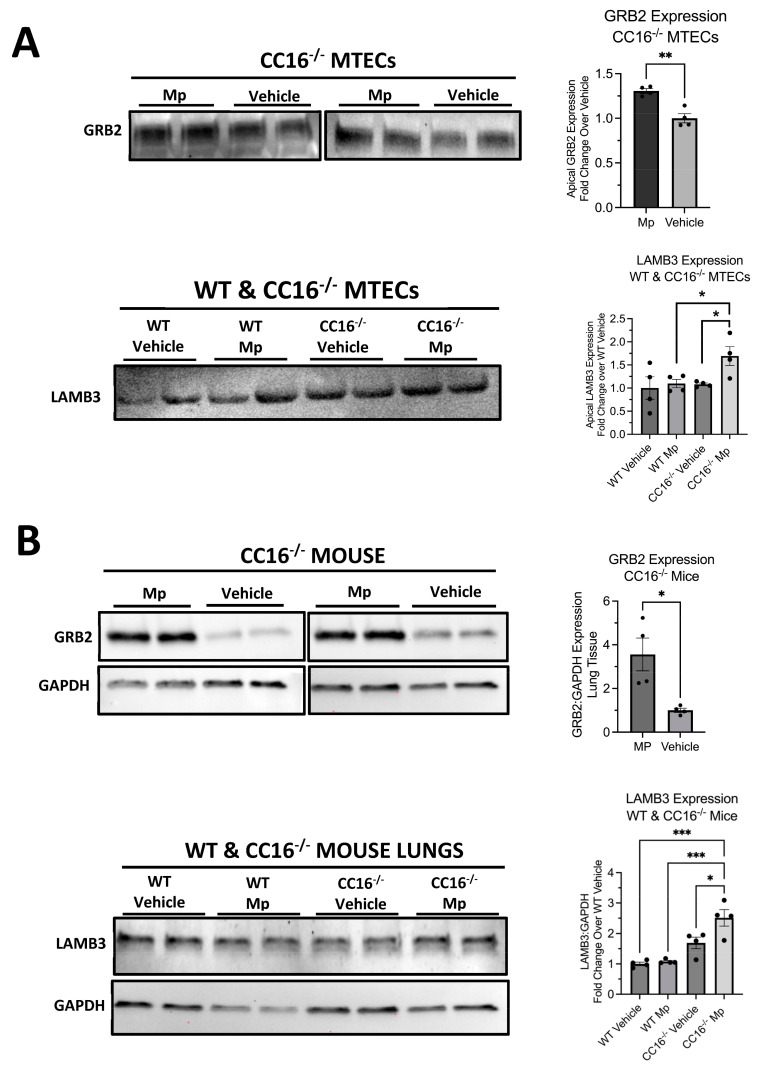
Validation of upregulated proteins by Western blot. The expression of GRB2 and LAMB3 was verified in WT and CC16^−/−^ MTEC apical supernatants (**A**) and mouse lungs (**B**). Representative blots are shown. GAPDH was used as a housekeeping control. Significance (* *p* < 0.05, ** *p* < 0.01, *** *p* < 0.001) was obtained by either unpaired *t*-test or one-way ANOVA Tukey’s multiple comparison test. Data are presented as mean ± SEM.

To ensure that the abundance and significance of proteins identified by mass spectrometry were reproducible, we validated two proteins, GRB2 and LAMB3, by Western blotting (Figure 7). Using mass spectrometry, we observed that GRB2 was upregulated by CC16^−/−^ MTECs during Mp infection compared to vehicle-treated CC16^−/−^ MTECs (Figure 4F); GRB2 was not detected from WT MTECs. Additionally, we observed that LAMB3 was upregulated by CC16^−/−^ MTECs during Mp infection compared to vehicle-treated CC16^−/−^ MTECs (Figure 4C,I) and Mp-infected WT MTECs (Figure 6C,G). The expression of GRB2 and LAMB3 was first validated in the apical secretions from WT and CC16^−/−^ MTECs (Figure 7A). In the apical secretions, GRB2 expression was significantly increased from Mp-infected CC16^−/−^ MTECs compared to vehicle-treated CC16^−/−^ MTECs (Figure 7A). Additionally, LAMB3 expression was significantly increased by Mp-infected CC16^−/−^ MTECs compared to vehicle-treated CC16^−/−^ MTECs and Mp-infected WT MTECs (Figure 7A). These results from the apical secretions recapitulate our observations from the mass spectrometry data.

To further validate our results in vivo, we measured the expression of GRB2 and LAMB3 in saline-treated and Mp-infected WT and CC16^−/−^ mouse lungs by Western blotting (Figure 7B). We obtained similar results to what we observed in our mass spectrometry data and apical secretion Western blotting. GRB2 expression was significantly increased from the lungs of Mp-infected CC16^−/−^ mice compared to vehicle-treated CC16^−/−^ mice (Figure 7B). Additionally, we observed that LAMB3 expression is significantly increased in lungs from Mp-infected CC16^−/−^ mice compared to vehicle-treated WT andCC16^−/−^ mice as well as Mp-infected WT mice (Figure 7B). Overall, these in vivo results further confirm our mass spectrometry data.

## 3. Discussion

CC16 has been shown to protect against the development of obstructive lung disease, in part due to its anti-inflammatory and antioxidant activities, during infectious and non-infectious conditions [9,35,36,37]. Despite the known biological activities of CC16, the mechanisms by which CC16 exerts these activities, especially regarding the pulmonary epithelium, have not been fully elucidated. To better understand how CC16 regulates pulmonary epithelial-driven responses during Mp infection, we utilized mass spectrometry and quantitative proteomics to identify apically secreted proteins from vehicle-treated and Mp-infected WT and CC16^−/−^ MTECs. Using this approach, we observed that during Mp infection, WT MTECs downregulate apical protein secretion; meanwhile, CC16^−/−^ MTECs upregulate apical protein secretion, with the majority of proteins being associated with airway remodeling. These results provide additional evidence that CC16 plays a vital role in protection from pulmonary epithelial-driven airway remodeling during Mp infection and are in line with our previous publications that demonstrate exacerbated airway remodeling mechanisms in CC16^−/−^ mice [20,21,38].

Previous clinical studies have shown that low serum and bronchoalveolar lavage fluid (BALF) CC16 levels are associated with decreased lung function in patients [22,23,25,39]. Regarding pulmonary infections, it has been shown that CC16 may, in part, exert its effects in the lungs by modulating susceptibility and responses to various respiratory infections [20,40,41]. Additionally, we have previously demonstrated that CC16^−/−^ mice and MTECs have significantly increased airway remodeling and Mp burden compared to their WT counterparts [20,21,38]. The work presented here supports these findings and further suggests that CC16 regulates pulmonary epithelial-driven responses during Mp infection. Based on this, as well as our previous work, the clinical implications for individuals with low CC16 levels, such as asthma, COPD, and cystic fibrosis patients [23,25,26,27,29,30,39,42], is that they may have chronic or persistent pathogen burden resulting in increased airway remodeling, which is in part due to pulmonary epithelial-driven responses.

To determine if CC16 regulates the expression of pulmonary epithelial-driven responses during Mp infection, we treated and infected WT and CC16^−/−^ MTECs with media (vehicle) or Mp, respectively, for 48 h, after which we collected and analyzed apically secreted proteins by mass spectrometry and quantitative proteomics. Using this method, we observed that CC16 has a significant impact on pulmonary epithelial responses during Mp infection: WT MTECs had less apical protein secretion during Mp infection (28 proteins) compared to vehicle treatment (1430 proteins). This downregulation by WT MTECs during Mp infection appears to be driven by a reduction in antioxidant responses compared to vehicle treatment, which is likely due to the reduction in host cellular metabolism that is often observed during Mp infection [18]. In contrast, CC16^−/−^ MTECs had the opposite effect: Mp infection in the absence of CC16 resulted in an increased secretion of apical proteins (911 proteins) compared to vehicle treatment (35 proteins). This increase in protein secretions during Mp infection may be driven by remodeling proteins, which is in line of our previous studies [20,21,38]. These data provide evidence that CC16 is likely a key factor regulating pulmonary epithelial-driven apical protein expression during Mp infection.

We further compared how WT and CC16^−/−^ MTECs respond to Mp infection, and from this analysis, we identified a total of 95 proteins that were differentially secreted by the two groups during infection. However, of the 95 total differentially secreted proteins, the WT MTECs only had two significantly increased proteins over the CC16^−/−^ MTECs, confirming that the presence of CC16 results in decreased apical protein secretion during Mp infection. The two proteins with increased secretion by the WT MTECs during Mp infection were CC16 itself and thrombin (THRB). Thrombin is a serine protease that is essential in platelet activation and blood coagulation [43]. Interestingly, thrombin has been shown to be present in airway surface liquid and increases upon respiratory virus infection [44]. Additionally, thrombin has been shown to stimulate IL-8 expression, via NFkB activation, due to respiratory infection and allergic asthma [45]. Furthermore, these data suggest that CC16 may protect the airway epithelium during Mp infection by essentially “shutting down” epithelial-driven responses that would activate the remodeling pathways compared to the CC16^−/−^ MTECs in which those pathways are activated.

Despite observing overall decreased apical protein secretion by the WT MTECs during Mp infection, we found that the CC16^−/−^ MTECs had increased overall protein secretion, therefore further implicating CC16 in regulating pulmonary epithelial-driven responses during Mp infection. Upon further analysis, the proteins that were upregulated by the CC16^−/−^ MTECs during Mp infection were predominantly associated with GO and KEGG pathway enrichment terms associated with the ECM and the cytoskeleton. The most significantly increased ECM protein was laminin beta 3 (LAMB3), which has been shown to be used as a receptor for *Pseudomonas aeruginosa* during respiratory infection [46]. We also observed several known basement membrane and actin proteins being apically expressed by the CC16^−/−^ MTECs during infection. We have several hypotheses as to why we observe this: (i) CC16 deficiency allows Mp to increase actin cytoskeletal reorganization; therefore, CC16 protects from cytoskeletal reorganization during infection; (ii) the increased abundance of actin and basement membrane components is a result of epithelial cell death caused by Mp infection, resulting in release of these components; therefore, CC16 protects from pulmonary epithelial cell death during Mp infection; and (iii) CC16^−/−^ MTECs have an increased apical expression of these ECM and cytoskeletal proteins, both of which may be related to airway remodeling, during Mp infection; therefore, CC16 protects from the epithelial-driven airway remodeling. Several studies have shown that Mp infection causes cytoskeletal reorganization [47] and cell death through oxidative stress [19,48]. Additionally, our group, along with several other groups, has shown that Mp infection increases the expression of airway remodeling proteins and genes in CC16^−/−^ mice and MTECs [20,21,49,50,51,52]. Based on this, there may be validity in each of our proposed hypotheses as to why we observe an increased expression of ECM and actin proteins by the CC16^−/−^ MTECs during Mp infection.

Since CC16 may impact these differentially expressed proteins at the level of cell secretion or gene expression, we therefore sought to determine if any of the genes encoding the differentially expressed proteins from the GO and KEGG enrichment analyses contained SNPs and whether these SNPs result in increased or decreased gene expression. Based on our GO and KEGG enrichment analyses, many of the proteins secreted by CC16^−/−^ MTECs during Mp infection may be associated with airway remodeling and bacterial invasion; therefore, the lack of CC16 upregulates the secretion of these proteins during Mp infection. We identified a variety of SNPs in the genes associated with these airway remodeling and bacterial invasion proteins and determined whether these SNPs result in increased or decreased expression of these genes, which may ultimately affect protein expression. For example, in Table 1, we show that the gene encoding for Growth Factor Receptor-Bound Protein 2 (*GRB2*) has a variety of SNPs that increase the expression of this gene, and possibly protein, related to airway remodeling. From a clinical perspective, if CC16 levels are decreased, such as in asthmatic patients [5,23,24,25,26], and these risk alleles for increased *GRR2* expression are present, this could result in significantly increased airway remodeling during Mp infection. Additionally, using two human datasets, we were able to assess whether genes associated with the proteins upregulated by Mp-infected CC16^−/−^ MTECs were associated with lung function and risk for asthma. We chose to use an asthma dataset since asthmatics have decreased CC16 levels [5,23,24,25,26] and are at increased risk for lung function decline during Mp infection [20,21,50,51,53,54]. Using these datasets, we identified that *GRB2* had variants that were significantly associated (*p* < 0.05) with a decline in forced expiratory volume in one second (FEV1; lung function) and/or an increased risk of asthma development. Several groups have shown the association between GRB2 and asthma [55,56,57,58]; however, we are the first to link increased GRB2 expression to CC16 deficiency, which may have an impact on asthma development, especially in those individuals prone to persistent Mp infections.

Overall, our data show that CC16 is an important regulator of pulmonary epithelial-driven responses during Mp infection. More specifically, during Mp infection, the presence of CC16 results in significantly decreased overall apical protein expression, which may protect the pulmonary epithelium, and lung in general, during times of infection from initiating remodeling pathways. A limitation of this study is the use of a single pathogen (Mp) at a single timepoint; therefore, additional studies should be performed to determine if these responses are pathogen specific or not and if pulmonary epithelial-driven responses are time-dependent over the course of infection. Also, CC16^−/−^ mice were generated several decades ago by common methods using a 129 mouse reference strain, and as such, they may have remnant 129 genetic material remaining after generations of backcrossing that could impact readouts [59,60]. Additionally, the analysis of pulmonary epithelial-driven responses using the MTEC model is a limitation, since this only focuses on mice; therefore, future studies will focus on the use of human nasal epithelial cells (HNECs) from healthy, CC16-sufficient individuals as well as asthmatics who have low CC16 levels. Despite this, these MTEC studies have provided us a strong foundational basis for our future experiments in HNECs. Future studies are needed to determine the mechanisms by which CC16 acts as a global suppressor of apical protein secretion by the pulmonary epithelium during Mp infection as well as the mechanisms by which Mp upregulates the expression of ECM and actin proteins in the absence of CC16. Taken together, our findings support the idea that CC16 plays a protective role within the pulmonary epithelium during Mp infection, which reinforces the potential of CC16 therapy as a novel approach to decrease pathogen burden and increase lung function in individuals with low CC16 levels, such as asthma, COPD, and cystic fibrosis patients [5,22,23,25,26,27].

## 4. Conclusions

We observed that the presence of CC16 results in decreased apical protein expression during Mp infection from MTECs. Conversely, a lack of CC16 during Mp infection results in an increased apical secretion of proteins that appear to be related to airway remodeling and enhance bacterial invasion in MTECS. Future studies will focus on understanding pulmonary epithelial-driven responses during Mp infection using HNECs as our human model system. Overall, our study provides evidence that CC16 is an important regulator of pulmonary epithelial-driven responses during Mp infection that likely plays a role in protection against airway remodeling.

## 5. Methods

### 5.1. WT and CC16^−/−^ Mice

All experiments were handled in accordance with the University of Arizona on IACUC-approved animal protocols. Lungs from ~6–8-week-old WT and CC16^−/−^ [60] mice on the C57BL/6J were obtained 3 days after Mp infection as previously described [20]. MTECs were isolated from a combination of male and female mice > 12 weeks of age for culture. All mice were born and raised in the same room in the University of Arizona Health Sciences animal facility and were tested to be specific-pathogen free according to standard protocols using sentinel mice from the same room.

### 5.2. Mouse Tracheal Epithelial Cell (MTEC) Isolation and Culturing

Tracheas were collected, and MTECs were obtained and cultured as previously described [20]. In short, tracheas were collected from WT and CC16^−/−^ mice [37], after which a longitudinal incision was made to obtain the epithelial cells from the mucosal lining of each trachea. The cells were collected in a 15 mL conical tube, centrifuged (900 rpm, 5 min, 4 °C), and resuspended in 5 mL Versene (Life Technologies; Waltham, MA, USA) for 15 min (37 °C). Next, the cells were centrifuged (900 rpm, 5 min, 4 °C), resuspended in Keratinocyte Serum-Free Media (KSFM) (Gibco; Waltham, MA, USA), and seeded for growth in T75 tissue culture-treated flasks (Corning; Corning, NY, USA).

In vitro MTEC expansion and culturing was based on the protocol published by Eenjes et al. [61]. In short, MTECs (375,000 cells) were seeded in T75 tissue culture-treated flasks and grown at 37 °C, 5% CO_2_ until 90% confluency was reached. Once confluent, MTECs were removed from the flasks and seeded onto Costar Transwell (12 mm, 0.4 μm membrane pores) 12-well plates at a density of 89,600 cells/Transwell. After seeding onto Transwells, the MTECs were incubated (37 °C, 5% CO_2_) for 48 h without changing the media. After the initial seeding period, the DMEM F-12 growth media on the apical and basal sides of the membrane was replaced every other day. When the cells reached 80% confluency (~1 week), the culture medium was replaced daily, only on the basolateral side, to establish an air–liquid interface (ALI).

### 5.3. Mp Infection for WT and CC16^−/−^ MTECs and Mice

Mp was purchased from ATCC (Manassas, VA, USA) (cat. no.: 15531) and grown in Remel SP4 broth (Thermo Fisher Scientific; Waltham, MA, USA) at 35 °C until adherent: approximately 4 passages [20]. Mp infection in MTECs was performed as previously described [20]. In short, after the apical surface of the MTECs was washed with sterile 1× PBS, Mp (1 × 10^6^ Mp/200 μL inoculum) was added to the apical side of the MTECs and incubated for 48 h (37 °C, 5% CO_2_). The infection time (48 h) and concentration (1 × 10^6^ Mp/200 L inoculum) was chosen based on our previous experiments showing CC16^−/−^ MTECs have significantly increased Mp burden compared to infected WT MTECs during those infection parameters [20].

WT and CC16^−/−^ mice were infected as previously described [20]. In short, Mp (1 × 10^8^ Mp/50 μL inoculum) was delivered via intranasal instillation while mice were under isoflurane anesthesia. Lung samples were harvested 3 days after infection, and lysates were processed for Western blotting.

### 5.4. Collection of Apically Secreted Proteins from MTECs

After vehicle treatment and Mp infection, 100 mL DMEM F-12 media (Gibco; Waltham, MA, USA) was added to the apical side of the MTECs. The MTECs were then washed 2× with the apical DMEM F-12 media to collect any apically secreted proteins adhered to the cell surface.

### 5.5. In-Solution Tryptic Digestion

In-solution tryptic digestion of the apically secreted proteins from WT and CC16^−/−^ MTECs was performed as described [62]. In brief, 100 mg of protein was subjected to acetone precipitation by adding six times the sample volume of pre-chilled 100% acetone and incubated one hour at −20° C. The precipitates were centrifuged at 16,000× *g* for 10 min at 4 °C and the acetone was removed. Then, 400 L of pre-chilled 90% acetone was added to the protein pellet, briefly vortexed and centrifuged at 16,000× *g* for 5 min at 4 °C. The remaining acetone was removed, and the protein pellets were air dried for 3 min, resuspended in 100 L of 50 mM NH4HCO3 and sonicated for 5 min. The samples were supplemented with dithiothreitol (DTT) at a final concentration of 5 mM and incubated at 70 °C for 30 min. Samples were cooled to room temperature for 10 min and incubated with 15 mM acrylamide for 30 min at room temperature while protected from light. The reaction was quenched with DTT with a final concentration of 5 mM and incubated in the dark for 15 min. One gram of Lys-C was added to each sample and incubated at 37 °C for 2–3 h while shaking at 300 rpm followed by the addition of 50 L of 50 mM ammonium bicarbonate and 2 g of trypsin and incubation overnight at 37 °C while shaking at 300 rpm. Then, 14.7 µL of 40% FA/1% HFBA was added to each sample and incubated for 10 min (final concentration is 4% FA/0.1% HFBA) to stop trypsin digestion. The samples were desalted with Pierce Peptide Desalting Spin Columns per the manufacturer’s protocol (Thermo Fisher Scientific, cat no. 89852), and the peptides were dried by vacuum centrifugation. The dried peptides were resuspended in 20 μL of 0.1% FA (*v*/*v*), and the peptide concentration was determined with the Pierce Quantitative Colorimetric Peptide Assay Kit per the manufacturer’s protocol (Thermo Fisher Scientific, cat no. 23275). Then, 350 ng of the final sample was analyzed by mass spectrometry.

### 5.6. Mass Spectrometry and Spectrum Count Data Processing

HPLC-ESI-MS/MS was performed in positive ion mode on a Thermo Scientific Orbitrap Fusion Lumos tribrid mass spectrometer fitted with an EASY-Spray Source (Thermo Scientific, San Jose, CA, USA) as previously described [63]. In brief, nanoflow liquid chromatography was performed without a trap column using a Thermo Scientific UltiMate 3000 RSLCnano System with an EASY Spray C18 LC column (Thermo Scientific, 50 cm × 75 μm inner diameter, packed with PepMap RSLC C18 material, 2 µm, cat. # ES803); loading phase for 15 min at 0.300 mL/min; mobile phase, linear gradient of 1–34% Buffer B in 119 min at 0.220 mL/min, followed by a step to 95% Buffer B over 4 min at 0.220 mL/min, hold 5 min at 0.250 mL/min, and then a step to 1% Buffer B over 5 min at 0.250 mL/min and a final hold for 10 min (total run 159 min); Buffer A = 0.1% FA/H_2_O; Buffer B = 0.1% FA in 80% ACN. All solvents were liquid chromatography mass spectrometry grade. Spectra were acquired using XCalibur, version 2.3 (Thermo Scientific). A “top speed” data-dependent MS/MS analysis was performed. Dynamic exclusion was enabled with a repeat count of 1, a repeat duration of 30 sec, and an exclusion duration of 60 s.

### 5.7. Label-Free Quantitative Proteomics

Progenesis QI for proteomics software (version 2.4, Nonlinear Dynamics Ltd., Newcastle upon Tyne, UK) was used to perform ion-intensity based label-free quantification as previously described [63,64,65,66]. In brief, in an automated format, .raw files were imported and converted into two-dimensional maps (*y*-axis = time, *x*-axis = *m*/*z*) followed by the selection of a reference run for alignment purposes. An aggregate data set containing all peak information from all samples was created from the aligned runs, which was then further narrowed down by selecting only +2, +3, and +4 charged ions for further analysis. The samples were then grouped in wild type versus knockdown. Peak lists of fragment ion spectra were exported in Mascot generic file (.mgf) format and searched against the Swissprot Mus musculus database (17,097 entries) using Mascot (Matrix Science, London, UK; version 2.6). The search variables that were used were: 10 ppm mass tolerance for precursor ion masses and 0.5 Da for product ion masses; digestion with trypsin; a maximum of two missed tryptic cleavages; variable modifications of oxidation of methionine and phosphorylation of serine, threonine, and tyrosine; 13C = 1. The resulting Mascot .xml file was then imported into Progenesis, allowing for peptide/protein assignment, while peptides with a Mascot Ion Score of <25 were not considered for further analysis. Progenesis QI for Proteomics is a software program that performs quantitative proteomics using extracted ion abundance. During experiment processing, Progenesis performs normalization to compensate for the variation between the different samples to avoid artifactual differences resulting from sample loading. Precursor ion-abundance values for peptide ions were normalized to all proteins. For quantification, proteins must have possessed at least one or more unique, identifying peptide. Principle component analysis and unbiased hierarchical clustering (heat map) was performed in Perseus [67,68] and volcano plots were generated in GraphPad Prism 8.0 (Graph Pad Inc.; San Diego, CA, USA). Apically secreted proteins from WT and CC16^−/−^ MTECs that had significant abundance were exported from Progenesis for further analysis. Using multiple cohorts (2 cohorts; *n* = 3/cohort), apically secreted proteins whose expression was reproduced in all cohorts based on the highest mean condition and significance were used for further analysis. Significantly secreted proteins (*p* < 0.05) were used input into the Database for Annotation, Visualization and Integrated Discovery (DAVID) [69] to identify enriched biological themes through the use of Gene Ontology (GO) as well as visualization of biological pathways using Kyoto Encyclopedia of Genes and Genomes (KEGG) pathway maps (https://david.ncifcrf.gov/, accessed on 17 April 2023).

### 5.8. Determination of GRB2 and LAMB3 Expression by Western Blotting

To determine GRB2 and LAMB3 protein levels in the lungs, right lung tissue from each sample was homogenized with 400 μL of RIPA (radioimmunoprecipitation assay) buffer (Teknova; Hollister, CA, USA) with protease inhibitors (Roche; Basel, Switzerland). After homogenization, the lungs were centrifuged at 12,000 rpm for 10 min at 4 °C, and the supernatant was collected. Protein concentrations from lung lysates were quantified using a Pierce BCA Protein Assay Kit (Thermo Fisher Scientific; Waltham, MA, USA). A standard concentration (10 μg/mL) of lung lysate was loaded onto Mini-Protean TGX precast gels (Bio-Rad Laboratories; Hercules, CA, USA). Antibodies for GRB2 (Invitrogen; Carlsbad, CA, USA) and LAMB3 (Invitrogen; Carlsbad, CA, USA) were used according to the manufacturer’s recommendations. All primary antibodies were diluted 1:1000 in 5% (*w*/*v*) nonfat dry milk-1X TBST and required an anti-rabbit secondary antibody (Cell Signaling; Danvers, MA, USA). The secondary antibody was diluted 1:2000 in 5% (*w*/*v*) nonfat dry milk-1X TBST. A ChemiDoc imaging system and Image Lab software (Thermo Fisher Scientific; Waltham, MA, USA) were used to image and quantify the densitometry of each Western blot.

## Figures and Tables

**Table 1:** SNP IDs and predicted expression changes for genes associated with select GO and KEGG enrichment proteins secreted by CC16^−/−^ MTECs during Mp infection.

Protein Name	Protein Abbreviation	Gene Abbreviation	SNP IDs in Lung Tissue ^a^	Effective Allele	*p*-Value	Predicted Expression Change Due to SNP within Lung Tissue ^b^
Heparin Sulfate Proteoglycan 2	PGBM	*HSPG2*	rs2229475	T	5.58 × 10^−14^	Increase
			rs115963344	T	4.84 × 10^−13^	Increase
			rs12724454	A	4.84 × 10^−13^	Increase
			rs12725566	A	4.84 × 10^−13^	Increase
			rs12737091	T	4.84 × 10^−13^	Increase
			rs12741617	A	4.84 × 10^−13^	Increase
			rs12742444	T	4.84 × 10^−13^	Increase
Adapter Molecule CRK	CRK	*CRK*	rs16946807	A	3.74 × 10^−8^	Decrease
			rs34490967	C	5.86 × 10^−8^	Decrease
			rs7208768	A	8.46 × 10^−8^	Decrease
			rs11652967	G	9.72 × 10^−8^	Decrease
			rs35316912	C	1.16 × 10^−7^	Decrease
Ras GTPase-Activating-Like Protein	IQGA2	*IQGAP2*	rs112872685	A	1.84 × 10^−7^	Decrease
			rs875541	A	9.95 × 10^−6^	Decrease
			rs10075621	T	1.40 × 10^−5^	Decrease
			rs4296785	T	1.42 × 10^−5^	Decrease
			rs4501323	C	1.42 × 10^−5^	Decrease
			rs4501324	C	1.42 × 10^−5^	Decrease
Syndecan-4	SDC4	*SDC4*	rs2267867	A	3.10 × 10^−8^	Increase
Laminin Subunit Beta 3	LAMB3	*LAMB3*	rs1534962	A	5.22 × 10^−14^	Decrease
			rs9429823	G	1.08 × 10^−12^	Increase
			rs2298928	T	2.05 × 10^−12^	Increase
			rs2298926	A	3.74 × 10^−12^	Increase
			rs1534962	A	5.63 × 10^−12^	Decrease
			rs9429823	G	5.12 × 10^−9^	Increase
Plexin-B2	PLXB2	*PLXNB2*	rs74933135	A	3.36 × 10^−8^	Increase
			rs117289563	T	1.05 × 10^−7^	Increase
			rs118067161	T	1.05 × 10^−7^	Increase
			rs142227764	T	1.05 × 10^−7^	Increase
			rs78854007	A	1.05 × 10^−7^	Increase
			rs146310944	T	1.05 × 10^−7^	Increase
			rs183166435	C	1.05 × 10^−7^	Increase
			rs117199604	A	2.33 × 10^−7^	Increase
Growth Factor Receptor-Bound Protein 2	GRB2	*GRB2*	rs12451296	A	3.33 × 10^−5^	Increase
			rs11649784	T	3.54 × 10^−5^	Increase
			rs12604003	A	3.54 × 10^−5^	Increase
			rs4789187	G	3.54 × 10^−5^	Increase
			rs9895739	T	3.66 × 10^−5^	Increase
			rs9909443	T	4.02 × 10^−5^	Decrease
			rs9901434	G	4.18 × 10^−5^	Increase
Dynactin Subunit 1	DCTN1	*DCTN1*	rs34215278	T	5.19 × 10^−16^	Decrease
			rs11555696	A	6.86 × 10^−16^	Decrease
			rs71418733	T	6.86 × 10^−16^	Decrease
			rs71418738	G	1.56 × 10^−15^	Decrease
			rs13021268	T	9.16 × 10^−15^	Decrease
			rs34988074	C	1.12 × 10^−14^	Decrease
			rs34522471	G	1.10 × 10^−11^	Decrease
			rs35241844	C	1.00 × 10^−9^	Decrease
Protein BRICK1	BRK1	*BRK1*	rs67342818	A	8.10 × 10^−9^	Decrease
			rs111284032	T	8.10 × 10^−9^	Decrease
			rs112180322	T	8.10 × 10^−9^	Decrease
			rs113378528	A	8.10 × 10^−9^	Decrease
			rs3774208	G	8.10 × 10^−9^	Decrease
			rs41464050	G	8.10 × 10^−9^	Decrease
			rs58862481	A	8.10 × 10^−9^	Decrease
Clathrin Light Chain B	CLCB	*CLTB*	rs13181024	T	2.97 × 10^−32^	Increase
			rs13168842	T	2.97 × 10^−32^	Increase
			rs13154366	A	2.19 × 10^−25^	Increase
			rs13175787	G	2.19 × 10^−25^	Increase
			rs13168952	A	5.90 × 10^−25^	Increase
			rs13180938	A	2.47 × 10^−23^	Increase
			rs13181024	T	1.94 × 10^−22^	Increase
			rs13168842	T	1.94 × 10^−22^	Increase
Integrin-Linked Protein Kinase	ILK	*ILK*	rs11826498	C	4.89 × 10^−15^	Increase
			rs11602107	A	1.17 × 10^−14^	Increase
			rs11605114	A	1.17 × 10^−14^	Increase
			rs2255405	A	1.56 × 10^−14^	Increase
			rs12451	T	2.62 × 10^−14^	Increase
			rs3741271	C	4.63 × 10^−14^	Increase
			rs2255538	A	9.88 × 10^−14^	Increase
			rs2292195	T	1.80 × 10^−13^	Increase

^a^ SNPs were identified in lung tissue using QTLbase (www.mulinlab.org/qtlbase/index.html) accessed on 17 April 2023 [32]. ^b^ Data used for the analyses described in the manuscript were obtained from the GTEx portal (www.gtexportal.org) accessed on 17 April 2023.

**Table 2:** Effect of GRB2 SNPs on lung function and asthma risk.

Gene	SNP ID	Risk Allele	Expression Change	Effect of SNP on Phenotype	*p*-Value
*GRB2*	rs9901434	G	↑	↓ FEV1	6.53 × 10^−6^
				↑ asthma risk	2.33 × 10^−2^
	rs9895739	T	↑	↓ FEV1	4.26 × 10^−6^
				↑ asthma risk	3.10 × 10^−2^
	rs11649784	T	↑	↑ asthma risk	2.22 × 10^−2^
	rs12604003	A	↑	↑ asthma risk	2.13 × 10^−2^
	rs4789187	G	↑	↑ asthma risk	2.22 × 10^−2^

## Data Availability

All data will be available upon acceptance of this manuscript.
